# Supplementary material for: Impact of Probiotic Combination in InR[E19]/TM2 Drosophila melanogaster on Longevity, Related Gene Expression, and Intestinal Microbiota: A Preliminary Study
Source: Microorganisms. 2020 Jul 11;8(7):1027. doi: 10.3390/microorganisms8071027 (PMC7409141; doi:10.3390/microorganisms8071027)
Supplement: Supplementary file 1 [file microorganisms-08-01027-s001.pdf]

Table S1. Primer Sequences of qRT-PCR used in this study.

Gene	Primer sequences (5'→3')	Reference
Cat	CCTCTGATTCCTGTGGGCAA; GACGACCATGCAGCATCTTG	[1]
sod2	AATTTCGCAAACTGCAAGC; TGATGCAGCTCCATGATCTC	[2]
gstd2	CTCCAATGTCTCCAGGTGGT; CCCAGTTCTCATCCCATCC	[2]
gclc	CGAGGAGAATGAGCTGTTCC; ACCAGACCCGGAAAAACG	[2]
Dro	CCATCGAGGATCACCTGACT; CTTTAGGCGGGCAGAATG	[2]
bsk	CACTCAGCAGGAATTATTCACAGA; TTAGAGTGCAGTCGGCCTTT	[3]
Sir2	CCGTTACTGAGGAGGAGCTG; GTAGATCGCACACGTCCTTG	[4]
Relish	GACCCGAAAGCTCGGCGCAAA; TCGCTCACGAGTTGCGAGCAA	[5]
upd3	ACTGGGAGAACACCTGCAAT; GCCCGTTTGGTTCTGTAGAT	[6]
dIlp5	CAAACGAGGCACCTTGGG; AGCTATCCAAATCCGCCA	[3]
dIlp2	ATCCCGTGATTCCACACAAG; GCGGTTCCGATATCGAGTTA	[3]
dfoxo	TCGAGTGCAATGTCGAGGAG; AGCGGTATATTGATGTCCAGCAG	[3]
InR	AACAGTGGCGGATTCGGTT; TACTCGGAGCATTGGAGGCAT	[2]
S6K	TGACCTAGAACCGGAATTGTG; TCCTCGCAGAGCTGTATGG	[3]
Tor	GCTCAGAGGCGAGAGACAAG; CCAGCTCACGGAGGATAAAG	[3]
4E-BP	CCAGATGCCCGAGGTGTA; AGCCCGCTCGTAGATAAGTTT	[3]
chico	GGCATACGGGCAGCTAGAC; TTCTTGAGGTAGCCACTCAGC	[3]
Imp-L2	GCCGATACCTTCGTGTATCC; TTTCCGTCGTCAATCCAATAG	[2]
E74B	GAATCCGTAGCCTCCGACTGT; AGGAGGGAGAGTGGTGGTGTT	[2]
Rp49	GACGCTTCAAGGGACAGTATCTG; AAACGCGGTTCTGCATGA	[8]

References

1. Staats, S.; Wagner, A.; Kowalewski, B.; Rieck, F.; Soukup, S.; Kulling, S.; Rimbach, G., Dietary resveratrol does not affect life span, body composition, stress response, and longevity-related gene expression in *Drosophila melanogaster*. *International Journal of Molecular Sciences* **2018,** *19 (1)*, 223.
2. Obata, F.; Fons, C.O.; Gould, A.P., Early-life exposure to low-dose oxidants can increase longevity via microbiome remodelling in *Drosophila*. *Nature Communications* **2018,** *9*, (1), 975.
3. Boyd, O.; Weng, P.; Sun, X.; Alberico, T.; Laslo, M.; Obenland, D.M.; Kern, B.; Zou, S., Nectarine promotes longevity in *Drosophila melanogaster*. *Free Radical Biology and Medicine* **2011,** *50 (11),* 1669-1678.
4. Tinkerhess, M.J.; Healy, L.; Morgan, M.; Sujkowski, A.; Matthys, E.; Zheng, L.; Wessells, R.J., The *Drosophila* PGC-1α homolog spargel modulates the physiological effects of endurance exercise. *PLoS ONE* **2012,** *7 (2),* e31633.
5. Bandarra, D.; Biddlestone, J.; Mudie, S.; Muller, H.A.; Rocha, S., Hypoxia activates IKK–NF-κB and the immune response in *Drosophila melanogaster*. *Bioscience Reports* **2014,** *34*, (4).

6. Woodcock, Katie, J.; Kierdorf, K.; Pouchelon, Clara, A.; Vivancos, V.; Dionne, Marc, S.; Geissmann, F., Macrophage-derived upd3 cytokine causes impaired glucose homeostasis and reduced lifespan in *Drosophila* fed a lipid-rich diet. *Immunity* **2015,** *42 (1)*, 133-144.
7. Westfall, S.; Lomis, N.; Prakash, S., Longevity extension in *Drosophila* through gut-brain communication. *Scientific Reports* **2018,** *8 (1)*, 8362.
8. Storelli, G.; Defaye, A.; Erkosar, B.; Hols, P.; Royet, J.; Leulier, F., *Lactobacillus plantarum* promotes *Drosophila* systemic growth by modulating hormonal signals through TOR-dependent nutrient sensing. *Cell Metabolism* **2011,** *14 (3)*, 403-414.
